# Novel *GLA* Variants: 10 Experts’ Tips for the Clinical Assessment of Fabry Disease

**DOI:** 10.3390/jcm15197655

**Published:** 2026-10-02

**Authors:** Federico Pieruzzi, Renzo Mignani, Sandro Feriozzi, Marialuisa Zedde, Gianni Carraro, Cristina Chimenti, Antonio Pisani, Marco Spada, Amelia Morrone

**Affiliations:** 1 Nephrology, Fondazione IRCCS San Gerardo dei Tintori, 20900 Monza, Italy; 2 School of Medicine and Surgery, University of Milano-Bicocca, 20126 Milan, Italy; 3 Department of Medical and Surgical Sciences (DIMEC), Alma Mater Studiorum University of Bologna, 40126 Bologna, Italy; renzo.mignani2@unibo.it; 4 Nephrology Department, IRCCS S. Orsola Hospital, 40138 Bologna, Italy; 5 Nephrology, Fondazione Policlinico Universitario Campus Bio-Medico, 00128 Rome, Italy; sandro.feriozzi@gmail.com; 6 Neurology Unit, Stroke Unit, Azienda Unità Sanitaria Locale-IRCCS di Reggio Emilia, 42122 Reggio Emilia, Italy; marialuisa.zedde@gmail.com; 7 Nephrology, Dialysis and Transplant Unit, Department of Medicine, Padova University Hospital, 35128 Padova, Italy; giannicarraro63@gmail.com; 8 Department of Medical and Cardiovascular Sciences, La Sapienza University of Rome, 00185 Rome, Italy; cristina.chimenti@uniroma1.it; 9 Department of Public Health, Federico II University of Naples, 80138 Naples, Italy; antonio.pisani13@gmail.com; 10 Department of Pediatrics, University of Turin, 10124 Torino, Italy; marco.spada@unito.it; 11 Laboratory of Molecular Genetics of Neurometabolic Diseases, Department of Neuroscience and Human Genetics, Meyer Children’s Hospital IRCCS, 50139 Florence, Italy; amelia.morrone@meyer.it; 12 Department of Neurosciences, Psychology, Drug Research and Child Health (NEUROFARBA), University of Florence, 50139 Florence, Italy

**Keywords:** Anderson–Fabry disease, α-galactosidase A, gene variant

## Abstract

The increasing use of next-generation sequencing (NGS), including multigene panels and exome and genome sequencing, has led to increased identification of rare and previously unreported variants in Mendelian disorders, though evidence supporting their clinical interpretation remains limited. In Fabry disease (FD), this issue is particularly relevant because the interpretation of individual *GLA* gene variants contributes to establishing whether a variant supports the diagnosis, with an impact on treatment decisions. In fact, FD is characterized by a phenotypic heterogeneity depending on patients’ biological sex and the underlying *GLA* variant. This represents the main reason why integrating genetic data with phenotypic expression is challenging, and defining the pathogenic role of novel variants remains complex. Early discrimination between pathogenic and non-pathogenic *GLA* variants is essential. However, variant classification, a definitive diagnosis of FD, and the decision to start disease-specific treatment are separate steps. A variant of uncertain significance (VUS), an isolated biochemical abnormality or non-specific organ involvement, is not in itself sufficient to establish the diagnosis or justify enzyme replacement or chaperone therapy. In fact, the most severe cases of FD have a significantly compromised quality of life up to reduced life expectancy, especially with regard to cardiac and renal complications. In recent years, some *GLA* variants have been reclassified following investigation and clinical observations by multidisciplinary working groups, making it possible to better define if they are pathogenic or not. Regardless of the clinical scenario leading to the identification of a novel *GLA* variant (i.e., family screening, neonatal screening, clinical profile suggestive of FD, incidental finding), any genetic finding becomes relevant when it is complemented by laboratory, clinical and instrumental investigations that require a concerted effort among the many health care professionals involved in the diagnostic process. The present work summarizes the outcomes of a series of expert meetings with the aim to provide some shared and practical indications for managing the genetic report of a novel *GLA* variant in order to promptly start the most suitable diagnostic journey and assess corresponding phenotype and clinical characterization.

## 1. Introduction

Fabry disease (FD) (OMIM #301500) is a rare, X-linked lysosomal storage disorder (LSD) caused by pathogenic variants in the *GLA* gene located on chromosome Xq22.1 (reference transcript NM_000169.3, MANE Select; GRCh38) that lead to α-galactosidase A (α-Gal A) deficiency and systemic accumulation of globotriaosylceramide (Gb3) and globotriaosylsphingosine (lyso-Gb3), resulting in multisystem involvement, with clinical manifestations including skin and eye lesions, neuropathic pain, gastrointestinal issues, and life-threatening renal, cerebrovascular, and cardiovascular complications [1]. Prevalence estimates based on clinical ascertainment range from 1 in 40,000 to 1 in 170,000 [2], whereas newborn screening in a ten-year program covering 234,642 newborns in Tuscany found an overall incidence of 1 in 9776 and an incidence of the classic phenotype of 1 in 46,928 [3]. Because these programs are based on enzyme activity, they identify essentially hemizygous males and miss most heterozygous females. The highly variable phenotypic expression is influenced by factors such as biological sex and the specific *GLA* variant, which can lead to either classic or late-onset subtypes [4,5]. To date, 1250 variants have been reported in the *GLA* gene (HGMD Professional, release 2026.2, accessed August 2026). Most are private, occurring in a single family or in a small number of families, and genotype–phenotype correlations are not always clearly established [6]. FD male patients often exhibit a more severe clinical course. However, heterozygous females with the same *GLA* variant should not be described merely as carriers: many develop clinically significant disease, with a phenotype ranging from asymptomatic to involvement as severe as in males. This variability reflects random X-chromosome inactivation (lyonization), which makes every heterozygous woman a cellular mosaic; skewed inactivation is an additional and less frequent finding, reported in about 29% of women with FD, in whom residual α-Gal A activity, severity scores and the progression of cardiac and renal involvement differed according to the direction and degree of skewing [7]. Its value at the individual level is limited, since the proportion of skewed samples differs markedly between tissues, from 7% in mouth epithelial cells to 35% in blood; clinical severity, α-Gal A activity, and lyso-Gb3 levels do not differ according to the skewing pattern [8]. Current treatment options include enzyme replacement therapy (ERT) using agalsidase-α, agalsidase-β, and pegunigalsidase alfa, and oral chaperone therapy with migalastat, to mitigate the progression of organ damage [9]. Timely therapy initiation can significantly influence disease trajectories, thus prompt and accurate diagnosis is paramount [10,11]. However, treatment response varies across patients, highlighting the need for a deeper understanding of individual determinants of disease expression, starting from the underlying genetic variant [12]. A significant issue in FD is the interpretation of VUSs, which are *GLA* gene nucleotide variants whose association with the disease remains unclear and sometimes never before described [13]. According to the classification by the American College of Medical Genetics and Genomics (ACMG) and the Association for Molecular Pathology (AMP) in their joint consensus recommendation, genetic variants in Mendelian disorders are classified as pathogenic (P), likely pathogenic (LP), VUS, likely benign (LB), and benign (B) [14]. When a *GLA* variant is identified, a comprehensive evaluation is recommended in order to decipher its nature not only for guiding treatment or avoiding any useless therapy, but also for risk assessment as to other family members [15]. The term “novel” *GLA* variants refers to previously unreported alterations in the *GLA* gene, whereas the use of “*de novo*” refers to variants that have arisen in the affected individual and are not inherited from either parent; a de novo variant may therefore be novel or already known. Establishing de novo occurrence requires testing the mother of a male proband, or both parents of a female proband, with confirmed biological relationships; a negative maternal result on blood does not exclude germline mosaicism [14,16]. The topic of *GLA* variants in FD is rather controversial, with a number of them (e.g., p.D313Y, p.E66Q, and probably p.R118C) initially described as pathogenic and then reclassified as benign or likely benign, and some reported in ClinVar (https://www.ncbi.nlm.nih.gov/clinvar/ (accessed on 12 September 2026)) with conflicting interpretations. Their frequency in the general population is ancestry-dependent and does not consistently exceed the 1% threshold that defines a common polymorphism [17,18]. Nowadays, technologies such as NGS easily identify gene variants, supported by clinical, biochemical, and histopathological evidence in order to determine their clinical impact [19,20]. *GLA* variants can occur in a single or few families; moreover, epigenetic factors can increase variability and complicate the assessment of any genotype–phenotype correlations, and in vitro studies such as *GLA* expression assays are needed [21,22]. However, this type of testing is available only in a limited number of specialized research laboratories, which have to deal with an increasing workload due to more frequent systematic screening protocols of high-risk populations, such as cohorts with chronic kidney disease, cryptogenic stroke or unexplained left ventricular hypertrophy, and of newborns, leading to more frequent detection of *GLA* variants and an increasing need for standardized guidelines for their interpretation [23,24,25,26,27,28,29].

This work is not a formal consensus on the interpretation and management of individual GLA variants. It reports the outcome of a structured discussion among nine Italian experts in FD—five nephrologists, a cardiologist, a neurologist, a pediatrician and a laboratory geneticist—selected for their long-standing clinical and laboratory experience in national reference centers for FD. The panel met two times in person and two times online, and at the first meeting the participants discussed and unanimously approved the working method. The literature was searched in MEDLINE via PubMed and in ClinicalTrials.gov from database inception to 31 December 2025; references published after that date were added during the revision of the manuscript. A tip was retained when at least seven of the nine experts agreed with its final wording.

The aim of this publication is to provide shared practical indications, “tips”, as to which diagnostic path might be the best to accurately investigate the type of genetic variant and the related phenotype to support the classification of novel GLA variants or VUSs and the characterization of the phenotype of the individuals who carry them. When the variant and the phenotype support the diagnosis of FD, this process informs the decision to start disease-specific treatment, which should then not be delayed. The statements hereby presented are the foundation for a structured diagnostic algorithm shown as in the graphical abstract, to effectively and promptly manage GLA novel variants or VUSs in clinical practice.

## 2. The Issue of Gene Variant Classification

With the advent of NGS, genetic laboratories are performing more complex genetic testing, spanning genotyping, single genes, gene panels, exomes, genomes, transcriptomes and epigenetic assays for genetic disorders, with new challenges in sequence interpretation [30,31]. When classifying a variant, laboratories find valuable information in population and disease databases such as gnomAD, as well as in the published literature. Genetic reports often include clinical assertions provided by physicians, while the ClinVar database stores variants with clinical observations and tracking of review status to enable more transparency [32]. Allele frequencies should be read with ancestry-specific counts, and the phenotypic classification issued by the Fabry disease genotype–phenotype workgroup provides a disease-specific reference for *GLA* variants [4]. Re-analysis of variants is a continuous process: previous gene classifications are constantly modified, and the availability of large population variant frequency data has led a few of them to be re-classified [33]. Optimal results are realized when referring healthcare providers and laboratories work collaboratively, and accurate and complete clinical information sharing is essential for the interpretation of genome-level DNA sequence findings [34]. For tests that cover a broad range of phenotypes (i.e., large panels, exome and genome sequencing), laboratories may find candidate causative variants and further follow-up with physicians for additional evidence to support the classification [33]. Testing other family members is a key step to establish if a variant is *de novo* or it co-segregates with the disease in the family [35]. In an X-linked gene such as *GLA*, segregation is informative mainly through hemizygous males, in whom genotype and phenotype are not confounded by X-inactivation. Collecting parental samples alongside that of the proband, i.e., trio testing, is recommended in the setting of exome and genome sequencing to document de novo occurrence, and in the absence of one or both parents, the inclusion of affected and unaffected siblings can be valuable [36,37]. In a ten-year newborn screening program in Tuscany, the investigation was extended to the mothers of all newborns carrying a pathogenic, likely pathogenic or uncertain *GLA* variant; all tested positive, so no de novo variant was identified [3]. A number of genetic variants result in a highly variable phenotypic expression and penetrance, further stressing the importance of providing comprehensive clinical data for their interpretation [38]. A pivotal issue is how to integrate genetic evidence into clinical decision-making; in newborns or adults, enzyme assays and laboratory tests, histological and physical findings, and imaging studies may allow variants to be reclassified [39]. Therefore, monitoring of the patient is recommended while clinical and instrumental evaluations are ongoing in order to use this information in clinical decision-making [38]. Another challenging issue related to variants’ definition and classification is that of allele frequency (AF), which cannot be reduced to a single cut-off. A rare AF does not on its own establish pathogenicity, since most rare variants in any gene are benign, and in the ACMG/AMP framework rarity contributes only at the supporting level (PM2_supporting). Conversely, the conventional 1% cutoff used to define a common polymorphism is not an appropriate benign threshold for a rare Mendelian disorder. Even the most frequent pathogenic *GLA* variants occur well below 1%: p.(Asn215Ser), reported as the most frequent variant associated with the late-onset cardiac phenotype in Western populations, and c.640-801G>A, previously designated IVS4+919G>A and c.639+919G>A, are both late-onset rather than classic alleles. The same is true, in European populations, of the variants reclassified as benign or likely benign or still reported with conflicting interpretations, such as p.(Arg118Cys), p.(Ser126Gly), p.(Ala143Thr) and p.(Asp313Tyr) [11,40,41]. Frequency is also ancestry-dependent: p.(Glu66Gln), now regarded as a functional polymorphism rather than a disease-causing allele, reaches an allele frequency of 1.046% in Korean individuals [18]. Observed frequencies should therefore be compared with a disease- and gene-specific maximum credible allele frequency, derived from disease prevalence, penetrance, genetic heterogeneity and the largest contribution any single variant makes to the disease, computed on ancestry-specific data and separately for hemizygous males and heterozygous females [42]. In the absence of a ClinGen *GLA* specification defining gene-specific thresholds for BA1 and BS1, any threshold applied should be reported together with the assumptions from which it was derived. Some *GLA* alleles are better described as low-penetrance risk factors than as classic Mendelian disease-causing variants, and forcing them into the pathogenic-benign dichotomy misrepresents the evidence. However, since FD is considered underdiagnosed, excluding a variant as non-pathogenic solely based on high AF may be misleading. Once a missense variant has been recognized as being pathogenic, the next step is to determine its association with a specific phenotype. The number of gene variants responsible for classic FD is far superior to the number of those associated with late-onset phenotypes, which highly segregate within populations and consequently have a higher prevalence due to their overall lower morbidity and minimal effects on genetic fitness [4,43]. No ClinGen Variant Curation Expert Panel specification is currently available for *GLA*, although the Lysosomal Diseases VCEP has released gene-specific specifications of the ACMG/AMP criteria for autosomal lysosomal disorders [44]. Curation of *GLA* variants therefore rests on the general 2015 ACMG/AMP framework, which was not designed for an X-linked lysosomal disorder with reduced age- and sex-dependent penetrance, integrated case by case with disease-specific evidence. The p.(Ala143Thr) variant deserves specific mention. Initially considered pathogenic for late-onset FD, it is now more consistently interpreted either as non-disease-causing or as an allele with markedly reduced penetrance, since hemizygous males frequently show substantial residual α-Gal A activity and normal plasma lyso-Gb3; isolated reports have nonetheless described left ventricular hypertrophy in hemizygous males and heterozygous women, and ClinVar currently records conflicting interpretations of pathogenicity [38]. It therefore requires individual reassessment rather than assignment to either category by default. In the Tuscan newborn screening cohort, p.(Ala143Thr) was the most frequent *GLA* variant identified, found in 19 of the 21 newborns carrying a benign variant, all of whom had normal lyso-Gb3 levels [3]. Enzyme-based screening is further affected by alleles that reduce measured α-Gal A activity without causing disease. p.(Asp313Tyr) is the paradigmatic example: the mutant enzyme is unstable at the neutral pH of plasma and yields a pseudodeficient plasma activity, while activity in leukocytes remains close to normal [17]. A discordant plasma-leukocyte result should therefore prompt consideration of a pseudodeficiency allele before a diagnosis of FD is entertained. For a novel variant, expression in a heterologous system such as HEK-293 cells measures the residual activity of the mutant enzyme and can be decisive for reclassification, contributing as PS3 or BS3, provided the assay is validated [14,45]. The same cell system is used to test amenability to migalastat, but the two questions are different: amenability asks whether the mutant enzyme responds to the chaperone, not whether the variant causes disease. When a variant lies at or near a canonical splice site, or when a deep intronic variant is identified, its effect on splicing cannot be inferred from the genomic sequence alone: RNA analysis of patient material, or a minigene assay where suitable RNA is not available, is the appropriate confirmatory test, as shown for the intronic variant described above [46]. Table 1 summarizes selected *GLA* variants whose pathogenicity assessment or associated phenotype has changed over time, together with the evidence supporting the revised interpretation.

## 3. Diagnostic Setting

To assess any genotype–phenotype correlation as to novel *GLA* variants, it is crucial to clarify the diagnostic context: *GLA* variants may represent the incidental finding of a gene/exome panel or a wider genome sequencing analysis to assess another Mendelian condition in an adult subject [57,58]; a different scenario is that of an adult or a newborn within a neonatal, prenatal, or familial screening, since they belong to an FD-positive or a *GLA* variant-positive family group [59]; finally, probands may have undergone genetic testing, since clinical signs/symptoms strongly suggestive of FD, such as angiokeratomas, cornea verticillata and others are present [60]. Depending on the context in which novel *GLA* variants emerge and in consideration of any already available data, the aim is to complete the diagnostic framework. If not already available, laboratory tests are initiated or repeated to verify the metabolic profile, and multi-organ or organ-specific clinical, histological, and instrumental evaluations are carried out in order to clarify the nature of the *GLA* variant. Moreover, the identification of novel *GLA* variants during newborn screening in asymptomatic subjects might be challenging, since random X-inactivation alone leaves most heterozygous women with enzyme activity within or close to the reference range, so that a normal maternal result carries no information about the variant; if no other male family member carries the same variant, the clinical assessment of these *GLA* variants relies on functional studies. In the organ-specific sections that follow, each finding is considered for what it adds to the interpretation of the variant, within an overall assessment of the clinical picture of the patient rather than as an individual grade. Findings with high specificity for FD, such as cornea verticillata, Gb3 storage with lamellar inclusions on biopsy once phenocopies have been excluded, and low native myocardial T1, strengthen the causal interpretation; non-specific findings, such as proteinuria, left ventricular hypertrophy, small fiber neuropathy, hearing loss, and gastrointestinal symptoms, support multiorgan involvement only when the genetic and biochemical data already point to FD.

*Experts’ Tips*

- *Verify whether the genetic test was performed following a clinical sign/symptom suggestive of FD, it was part of an extended or selective screening (i.e., prenatal, neonatal, familial), or it was due to an incidental finding during the genetic assessment of another Mendelian condition.*
- *Extend testing to relatives as directed by the pedigree and by the confirmed familial variant, rather than by a fixed degree of relatedness. Family members who test positive should follow the diagnostic procedure of the proband. Cascade testing also serves segregation analysis: failure of a GLA variant to co-segregate with the phenotype in several affected relatives is strong evidence in favor of benignity (BS4), whereas its presence in unaffected relatives, particularly women and young men, is not in itself evidence against pathogenicity, since penetrance is age- and sex-dependent, whereas co-segregation in multiple family members strengthens the evidence for pathogenicity (PP1); in an X-linked disorder, segregation is informative mainly through hemizygous males [14,37].*

## 4. Metabolic Profile

Preliminary laboratory testing can confirm or rule out suspicion of FD when a novel *GLA* variant is identified, and the assessment of metabolic profile is based on measurements of α-Gal A activity and lyso-Gb3 levels [61].

- The α-Gal A assay discriminates reliably only when activity is absent or markedly reduced. In hemizygous males, a severe deficiency supports the biochemical diagnosis, whereas normal leukocyte activity rules it out for the classic phenotype only: in late-onset variants, residual activity may fall within the lower reference range, and the result is sensitive to preanalytical and analytical variables. In heterozygous females, the assay has no exclusion value, since activity is frequently normal. Enzyme assay on a dried blood spot (DBS) is generally used for screening [62], whereas leukocytes are the reference matrix for confirmation; a plasma activity lower than the leukocyte activity should prompt consideration of a pseudodeficiency allele, as described above for p.(Asp313Tyr) [17]. Fibroblast assay is reserved for cases in which the result on blood is discordant or uninterpretable [61]. Residual activity in classic hemizygous males is generally below 5% of the mean reference value [5], whereas intermediate levels are described in late-onset variants; in heterozygous females, activity is frequently normal because of random X-chromosomal inactivation. Intermediate values should therefore not be read as a graded measure of disease risk.
- Unlike Gb3, lyso-Gb3 is a soluble molecule that is more readily excreted from cells [63]. Lyso-lipids are generated by acid ceramidase-mediated deacylation of the accumulating glycosphingolipid, a route described in other lysosomal glycosidase deficiencies as well [64]. In FD, the excess of Gb3 is converted into lyso-Gb3. Plasma lyso-Gb3 has been increasingly described as a sensitive biomarker of FD [65,66,67,68]. A few studies have pointed out that lyso-Gb3 levels correlate with cardiac disease severity [69,70]; it was reported that individuals with a novel *GLA* variant and organ involvement consistent with FD had lyso-Gb3 levels ≥2.7ng/mL, whereas in subjects with a novel *GLA* variant and no organ involvement levels were below that value [71]. The threshold comes from a single European cohort of 124 individuals with *GLA* variants, which separated 55 patients with classical FD from 17 carrying atypical variants, and it has not been validated as a general cut-off across laboratories: plasma is the matrix for clinical interpretation, the results are method-dependent and not interchangeable between laboratories [72], and reference intervals must be those of the reporting laboratory. As for the enzyme assay, plasma lyso-Gb3 is informative when clearly elevated; a normal or only mildly increased value does not exclude late-onset disease and has limited exclusion value in women, in whom levels overlap extensively with the reference range [57]. Moreover, lower levels of lyso-Gb3 at treatment initiation are correlated with better long-term outcomes [73,74], and cumulative exposure to lyso-Gb3 during prolonged untreated period appears to predispose to worse response to therapy [75].

*Experts’ Tip*

- *Carry out a α-Gal A assay on DBS for screening and on cells (i.e., leukocytes, fibroblasts) for confirmation; conduct lyso-Gb3 measurement in plasma for preliminary metabolic assessment of GLA variants.*

## 5. Renal Profile

In classic patients, Fabry nephropathy is related to a progressive decrease in renal function with deposition of Gb3 in all cell types (i.e., vascular endothelial cells, vascular smooth muscle cells, mesangial cells, interstitial cells, podocytes, and distal tubular epithelial cells) during fetal development [76]. Increased spot albumin/creatinine ratio (ACR) may already occur before 10 years of age, decline in glomerular filtration rate (GFR) along with hyperfiltration is seen from adolescence, progressive chronic kidney disease (CKD) with overt albuminuria and 24 h proteinuria is described in the second decade of life, and chronic renal insufficiency and end-stage renal disease (ESRD) develop between the third and fifth decades, leading to dialysis especially among classic male patients, while kidney dysfunction is less prevalent and milder among heterozygous females [77,78,79].

- When a *GLA* variant is identified, complete laboratory investigations of kidney function should be carried out, i.e., serum creatinine along with cystatin C to estimate GFR (eGFR), urinary protein excretion (spot ACR, PCR and 24 h proteinuria), and blood pressure measurement [80]. Assessment of proteinuria and GFR can be used for staging CKD, while urinary protein excretion is strongly associated with renal disease progression in FD, regardless of gender. In particular, a high value of eGFR (>135 mL/min/1.73 m^2^) should be accurately assessed in suspected hyperfiltration in a young individual, potentially irreversible histological changes can be observed in renal biopsies of children before the onset of albuminuria [81], and histological evidence of Gb3 accumulation as well as cell and vascular injury in renal tissue can be present prior to microalbuminuria and clinically significant renal events [82]. Therefore, although albuminuria and proteinuria are the most commonly used renal biomarkers; they show low sensitivity in identifying incipient nephropathy, and proteinuria may not be evident in patients with advanced kidney disease and does not necessarily correlate with GFR decline [83].
- Gb3 is elevated in the urine of FD patients, especially when no residual enzyme activity is present, while lower or normal levels are reported in female heterozygotes and in cohorts with residual enzyme activity [84]. Therefore, urine Gb3 is not an ideal biomarker for FD diagnosis, especially when a *GLA* variant is detected. On the other hand, albuminuria A2 and proteinuria (>150 mg/day), which are usually the first signs of renal involvement in FD, have been described as core biomarkers for diagnosis, evaluation of kidney damage and follow-up after therapy initiation, along with serum creatinine and cystatin C for eGFR assessment. Other renal biomarkers of early or subclinical renal involvement, such as beta 2-microglobulin (β2M), neutrophil gelatinase-associated lipocalin (NGAL), podocyturia, and urinary excretion of CD80 [78,85,86,87,88,89,90], could be evaluated in specific cases.
- Urine microscopy can highlight some basic findings for diagnosing and assessing FD progression, i.e., Maltese cross particles, urinary mulberry cells, and podocyturia, which can be useful in the overall assessment of a *GLA* variant, even though they are not pathognomonic of the condition [91,92,93]. Maltese cross particles are vacuolated epithelial cells filled with glycosphingolipids, visualized through polarized light microscopy [94]. Urinary mulberry cells are distal tubular epithelial cells in which Gb3 has accumulated, leading to a lamellar appearance, and they can be detected before kidney damage is identified [95]. Since the loss of urinary podocytes correlates with glomerular injury, it has been suggested as a potential diagnostic tool and a way to guide treatment strategies in FD nephropathy [96]. In FD patients with normal levels of albuminuria or proteinuria, the number of urinary podocytes can be significantly higher when compared to healthy individuals, suggesting that this biomarker, indicating a clinically silent kidney damage, may precede proteinuria [97]. Moreover, podocyturia is described as inversely related to GFR in male patients, and it is correlated with clinical severity in FD nephropathy, displaying a potentially prognostic value [78,98].
- Routinely, ultrasound (US) renal examination may detect parapelvic cysts (PCs), whose presence should prompt one to consider FD diagnosis, especially in subjects with an unclear family history of renal disease or when other stigmata of the disease are evident [99]. Even though the presence of PC is not currently regarded as a pathognomonic sign of FD and its pathogenesis is unclear, its prevalence in FD patients ranges from about 30% to 40%, compared with cohorts affected by other renal conditions or with the general population, in whom it is reported between 1% and 6% [99,100,101,102].
- Renal biopsy has become a valuable tool in complex genetic cases, especially when results of biochemical and molecular analyses are inconclusive [103]. Its indication should nonetheless be selective, since kidney biopsy carries a small but definite risk of complications, mainly bleeding, and is justified when the diagnosis remains uncertain after genetic and biochemical assessment, when kidney disease is atypical or a competing nephropathy is possible, or when histology is expected to change management. Kidney biopsy provides distinctive features to differentiate renal diseases, especially when clinical context, genetic data, and family history are unclear [104]. The International Study Group of Fabry Nephropathy (ISGFN) highlighted the role of kidney biopsy as part of the baseline assessment of FD nephropathy, as renal tissue involvement precedes clinical signs [95]. Early and prominent accumulation of Gb3 leads to cytoplasmic vacuolization and the presence of characteristic concentric lamellated inclusions (i.e., zebra bodies), predominantly observed in podocytes at electron microscopy [105]. Even though zebra bodies are hallmark features of FD, their identification necessitates a thorough evaluation to rule out other potential causes, such as rheumatoid arthritis, lupus nephritis, and hydroxychloroquine-chloroquine- and carbamazepine-induced phospholipidosis [105,106]. Proximal and especially distal tubular cells exhibit vacuolization due to Gb3 deposits, which are also found in glomerular endothelial and mesangial cells, contributing to glomerular dysfunction [107]. Segmental and global glomerulosclerosis are common findings even in early-stage CKD and correlate with disease progression and renal function decline as well as interstitial fibrosis and tubular atrophy [108]. Finally, Gb3 accumulation in vascular smooth muscle and endothelial cells leads to arteriolar hyalinosis along with myo-intimal cell proliferation and endothelial thickening, contributing to ischemic injury and hypertension, especially in male patients, leading to worse renal outcomes [109,110].

*Experts’ Tips*

- *Perform repeated measurements of (a) eGFR (check also for hyperfiltration) with creatinine and cystatin C; (b) albuminuria/proteinuria (ACR/PCR), preferred to timed collections, particularly in children, with 24 h proteinuria reserved to confirm positive spot samples in adults; (c) urinary sediment; and (d) renal ultrasound to check for the presence of PC.*
- *Consider kidney biopsy in subjects with renal involvement when the diagnosis remains uncertain after genetic and biochemical assessment, when kidney disease is atypical or a competing nephropathy is possible, or when histology is expected to change management, and carry out electron microscopy histological examination along with immunohistochemical investigation for Gb3 deposits. In asymptomatic subjects, evaluate whether to perform a kidney biopsy only in specific cases and based on available evidence of other FD-related organ involvement.*

## 6. Cardiological Profile

Lysosomal sphingolipid storage in FD occurs in all cardiac cell lines, i.e., myocytes, fibroblasts, conduction tissue, and endothelial, smooth muscular, and endocardial cells [111]. Cardiac involvement represents a leading cause of impaired quality of life (QoL) and mortality in both classic and later-onset FD, and the prevalence of FD in adult cohorts with unexplained left ventricular (LV) hypertrophy (LVH) ranges from 0.5 to 1% [112]. Cardiac damage remains subclinical for many years before FD symptom onset. While early extracardiac manifestations may suggest diagnosis in classic patients, in the late-onset cardiac phenotype, FD diagnosis may be more challenging, and FD cardiomyopathy needs to be differentiated from other more frequent forms of LVH [113].

- As to cardiovascular biomarkers, the amino terminal fragment of the brain natriuretic propeptide (NT-proBNP) has been studied as a diagnostic and prognostic predictor of heart disease in FD cohorts [114], while sphingosine-1-phosphate (S1P) was identified as a biomarker involved in cardiovascular remodeling in FD with a strong correlation with the LVM index. High-sensitivity cardiac troponin (i.e., hs-cTnI and hs-cTnT) serves as a sensitive blood biomarker for detecting and staging Fabry cardiomyopathy, correlating strongly with imaging markers of myocardial involvement, such as late gadolinium enhancement and left ventricular hypertrophy. Elevated troponin levels in FD patients reflect ongoing myocardial damage and fibrosis rather than acute coronary events, and a defined low-threshold hs-cTn (<8.5 ng/L) can reliably exclude significant cardiomyopathy. Serial measurements may therefore aid in early diagnosis, risk stratification, and monitoring of disease [115].
- Electrocardiogram (ECG) can show early indicators of FD [116]:
  - The PR interval is frequently altered in FD, and PR shortening can be detected in the initial phase of the disease, even with no LVH, while atrioventricular (AV) blocks are more frequent in the advanced phase of cardiac disease [117].
  - FD also affects the cardiac conduction system at all levels, from the sinoatrial node to the distal ramification of Purkinje fibers, leading to cardiac rhythm alterations. The mechanisms of bradyarrhythmia in FD are not completely understood, but post-mortem histological studies described the fibrosis and apoptosis of cardiac conduction tissue [118]. On the other hand, factors such as progressive Gb3 and lyso-Gb3 accumulation, atrial remodeling, and diastolic dysfunction may be linked to atrial fibrillation (AFib), which is considered an intermediate-to-late FD complication [119].
  - Similar to PR interval, QRS modifications in FD are correlated with the early or advanced phase of the cardiac disease. During the initial phase of the disease, acceleration of intra-ventricular conduction and QRS narrowing could be observed, whereas in later disease stages a progressive degeneration of the myocardial conduction system takes place, leading to QRS prolongation [120]. Other studies demonstrated right bundle branch block (RBBB) more commonly than left bundle branch block (LBBB) in FD patients; the reasons for the peculiar susceptibility of the right bundle branch are unclear [121].
  - FD-related ECG abnormalities also involve LV repolarization, and studies reported ST segment and T wave alterations as frequent findings in these patients [122].
- Bidimensional (2D) echocardiography and cardiac magnetic resonance (CMR) could provide further evidence to confirm or rule out FD diagnosis [123,124,125,126]. Severe LVH in female patients usually develops later, replacement fibrosis can be identified by CMR before hypertrophy appears, and according to the Fabry Outcome Survey (FOS) about 46% of female FD patients develop cardiac symptoms [127,128]. In late-onset FD, cardiomyopathy is often the only manifestation, and LVH (i.e., LV maximum wall thickness ≥ 12 mm in males) is typically found at echocardiography [124]. Although diffuse concentric LVH is the most prevalent finding, regional LVH of the septum may cause dynamic LV outflow tract obstruction (LVOTO), with systolic anterior motion of the mitral leaflet [128]. Other presentations include mid-ventricular obstruction, papillary muscle hypertrophy, and distal LVH, while hypertrophy of the right ventricle (RV) and aortic root dilation with preservation of ejection fraction are also ultrasound FD-related findings [129,130,131]. The prevalence of aortic dilatation in FD increases with age due to degenerative changes of the aortic media caused by glycolipid accumulation in the endothelium [132]. The prevalence of RV hypertrophy (RVH), i.e., wall thickness > 5 mm, ranges between 40% and 70% in FD, affecting males and females equally, and correlates to disease severity [133].
- CMR, including late gadolinium enhancement (LGE) and T1 mapping, is advised for further characterization of myocardial tissue in high-risk patients with *GLA* variants. Moreover, CMR allows assessment of FD structural changes at the inferolateral wall and anteroseptum as well as early detection of increased myocardial trabeculation, left atrial dilatation, RVH, and impairment of atrial deformation and compliance likely caused by Gb3 accumulation [134]. LGE imaging enables the detection of myocardial replacement fibrosis, showing a mid-wall distribution in the basal-infero-lateral area of the LV, which should raise FD suspicion in patients with unexplained LVH [135]. The prevalence of LGE ranges between 40% and 65%; it correlates with the severity of LVH in male FD patients and may precede the development of LVH especially in women. LVH has been described in other conditions, i.e., hypertension and aortic stenosis, hypertrophic cardiomyopathy, and cardiac amyloidosis; therefore, substantial value to FD differential diagnosis is added by mapping, in which individual image pixels are color-coded for longitudinal (T1) or transverse (T2) magnetism [136]. FD typically lowers myocardial native T1 because of intracellular glycosphingolipid storage, with a concentric T1 attenuation pattern in the early phases of the disease before LVH is detected [137,138]. Indeed, reduced T1 is described in approximately 40% of LVH-negative patients and correlates with early electrocardiographic changes and disease progression, becoming prominent in the advanced phases of the condition. However, the signal may become weaker with inflammation and fibrosis and even reverse in the presence of extensive LGE; thus, T1 mapping should be interpreted with caution [139]. T2 is typically elevated in areas of LGE, and it correlates with troponin rise in FD, supporting the role of inflammation in FD cardiomyopathy. In line with positron emission tomography (PET) and endomyocardial biopsy (EMB) findings, Gb3 accumulation leads to myocardial inflammation, detected by histological examination in up to 56% of patients [140,141].
- EMB findings in FD show that myocyte histological changes precede imaging findings: cardiomyocyte vacuolization is observed before LVH, Gb3 storage occurs before T1 lowering, and fibrosis is described before LGE [124,142]. EMB is, however, invasive and subject to sampling error and should be reserved for selected patients with a GLA variant and cardiac symptoms, i.e., angina, dyspnea, and palpitations, in whom non-invasive evaluation including CMR is inconclusive, as in left ventricular hypertrophy with a GLA variant of uncertain significance, high residual α-Gal A activity or low lyso-Gb3; the result is expected to change management, which should be performed in specialized centers [143,144].

*Experts’ Tips*

- *Perform a complete cardiological evaluation with measurement of cardiac biomarkers, including evaluation of heart function by ECG, Holter, echocardiogram, and CMR, with T1 and T2 mapping to check for evidence of FD signs.*
- *Consider endomyocardial biopsy, in specialized centers, in patients with cardiac signs or symptoms suggestive of FD when the heart appears to be the only organ involved, non-invasive evaluation including CMR is inconclusive, and the result is expected to change management.*

## 7. Neurological Profile

The neurological involvement of FD refers to both the peripheral (PNS) and central nervous system (CNS), and the latter includes the VIII cranial nerve and the neuro-enteric plexus.

- PNS involvement in FD presents as a ganglionopathy due to Gb3 accumulation in the dorsal root ganglion neurons and Schwann cells, which causes loss of cell functions with secondary degeneration of the afferent bundles of nerve fibers in their distal site. Studies have shown reduction of both myelinated and non-myelinated fibers as well as decreased intraepidermal nerve fiber density [145,146]. Small fiber neuropathy (SFN) in FD does not display specific features and involves autonomic fibers, responsible for acroparesthesias, neuropathic pain, abnormal thermal sensation, impaired sweating, and GI dysmotility [147]. The VIII cranial nerve is also involved, leading to hearing loss and imbalance [148]. Fabry-related neuropathic pain due to SFN is often the earliest manifestation of FD in children, being described in boys as early as 3 years of age or less and later on in girls [149]. Fabry crises are characterized by an agonizing burning sensation originating in the acral segments of the limbs, radiating in a distal-to-proximal direction, which may be precipitated by an increase in body temperature due to exercise, fever, and warm environments, secondary to impaired sweating [150]. In adulthood, pain is usually less severe, even though its prevalence is described as high as 80% and heavily conditioned by comorbidities, e.g., chronic kidney disease [151]. However, FD is only one among multiple causes of SFN, and in patients without any systemic involvement a pure SFN phenotype, it never leads to a straightforward FD diagnosis [152,153].
- CNS involvement in FD includes a more commonly reported cerebrovascular phenotype, due to the damage in the endothelial lining and in the smooth muscle cells of the tunica media and a less frequently described direct neuronal involvement, showing a prodromal neurodegenerative clinical phenotype [154]. Cerebrovascular manifestations in FD may include both ischemic and hemorrhagic stroke, as well as chronic small vessel disease (SVD) without any acute stroke; the large artery involvement usually presents as dilatative arteriopathy, which is also a rather common finding in other monogenic diseases and in the general population due to ageing and other risk factors [155,156,157]. Neuroradiological findings of FD on magnetic resonance imaging are non-specific and include white matter hyperintensities (WMHs) with variable severity degree, small vessel infarcts (SBI), lacunae, enlarged perivascular spaces (EPVs), and cerebral microbleeds [158].

*Experts’ Tips*

- *Carry out a thorough case history to assess the presence of other systemic signs and symptoms of FD and a complete neurological examination to select the subjects to be referred to further specific instrumental investigations.*

## 8. Systemic Signs and Symptoms

Systemic signs and symptoms sometimes represent the earliest manifestations of FD and can be the reason for genetic assessment, or they can help to decipher genetically unclear results in subjects who present cardiac, renal or neurological findings raising FD suspicion.

- Absence and decreased ability to sweat, i.e., anhidrosis and hypohidrosis, which leads to hyperthermia, poor exercise tolerance, and altered fever manifestation, are reported in 53% of classic males and 28% of females with FD. Sweating impairment is due to sympathetic sudomotor fiber dysfunction, and it can be measured by means of Quantitative Sudomotor Axon Reflex Tests (QSARTs) [159].
- Auditory and vestibular abnormalities due to involvement of cranial nerve VIII are frequently reported in the classic phenotype of FD, ranging from tinnitus to vertigo and, in particular, hearing impairment (hypoacusis), which has been described as an early indicator of undetected FD in females and otherwise asymptomatic individuals, although these complaints are very common in the general population [160].
- Gastrointestinal (GI) dysfunction is common in FD, and it is associated with deposition of Gb3 in the autonomic ganglia and mesenteric blood vessels of the gut [161]. GI symptoms include abdominal pain, bloating, diarrhea, constipation, nausea, and vomiting, as well as failure to gain weight, and often appear early in classic patients [162]. In order to carry out differentiation with Irritable Bowel Syndrome (IBS), techniques such as standard and video capsule endoscopy, scintigraphy, and intestinal biopsy can be used [163]. Radiographic studies may reveal thickened, edematous colonic folds, mild dilatation of the small bowel, granular-appearing ileum, and the loss of haustral markings throughout the colon [164].
- Ophthalmological signs in FD occur as early as the first decade of life, and patients can display cornea verticillata and Fabry cataract, as well as increased retinal vessel tortuosity, conjunctival telangiectasias and aneurysm-like formations, but none of these signs affects visual function [165]. Cornea verticillata (i.e., whorl-like, linear pigmentation in the inferior part of the cornea, observed by slit-lamp microscopy) is considered a pathognomonic sign of FD occurring in the majority of classical males, even though therapy with amiodarone or chloroquine can result in similar findings [166]. Fabry cataract is characterized by posterior capsular whitish spoke-like deposits of granular material, and it is considered to be another pathognomonic sign of FD, even though mannosidosis might cause similar lens opacities, while increased ocular vessel tortuosity has also been reported in fucosidosis as well as in healthy individuals [167]. Due to the early onset of ocular signs, ophthalmologists have the opportunity to identify FD before the condition is advanced; therefore, any patient with cornea verticillata or Fabry-like cataract, especially in combination with retinal vascular tortuosity, conjunctival vascular telangiectasia, or lenticular opacities, should undergo a comprehensive review of symptoms, family history and genetic testing, after a careful exclusion of other causes [168].
- Skin abnormalities have been reported in 78% of males and 50% of females with classic FD [169]. Angiokeratomas are the most common dermatological sign, occurring in 66% of males and in 36% of females with classic FD, even though they can also appear in other LSDs (i.e., mannosidosis, fucosidosis, sialidosis, b-galactosidase deficiency, and Schindler disease) [170]. Angiokeratomas are described as 1–5 mm, pinkish, dark red, blue-black, non-blanching macules or papules, which increase in number and size with age and appear on the umbilicus, hands, knees, elbows, and upper back and chest regions, especially in female subjects, spreading to the genitals mainly in males during adolescence [171]. Telangiectasia in sun-exposed areas is the second-most common dermatological sign in FD, appearing as small superficial angiomas due to cumulative damage of the vascular endothelial cells with vessel dilatation [172]. Although skin biopsy may be a useful additional diagnostic test, findings can be normal in heterozygous females [173]. Nonetheless, FD should be considered in patients when angiokeratoma corporis diffusum is accompanied by other dermatological signs, such as linear telangiectasia and sweating impairment [174].

*Experts’ Tips*

- *Assess the presence of early-onset systemic symptoms, e.g., GI alterations and acroparesthesias, and carry out a differential diagnosis.*
- *Assess the presence of early-onset pathognomonic or highly suggestive signs of FD, i.e., cornea verticillata and angiokeratomas, and of less specific signs that support multiorgan involvement, i.e., hypoacusis and hypohidrosis/anhidrosis.*

Table 2 summarizes the 10 Experts’ Tips.

## 9. Conclusions

The increasing detection of novel *GLA* variants makes it necessary to determine their nature, since their misinterpretation can lead to either missed opportunities for early treatment or unnecessary and potentially harmful interventions. Once the variant and the phenotype support the diagnosis, the timely start of disease-specific treatment in subjects with the most severe form of the condition makes it possible to slow down and limit damage to vital organs, considerably improving QoL; a variant of uncertain significance, an isolated biochemical abnormality or non-specific organ involvement does not in itself justify treatment. Furthermore, the identification of new *GLA* variants makes it possible to extend the tests to other family members.

This work has several limitations. The experts were selected by the authors and may not represent the full range of clinical practice; no formal consensus methodology, such as a Delphi process, was applied; the panel included only Italian centers; and the tips have not been validated prospectively. They should therefore be regarded as practical guidance to be tested in clinical practice.

Since FD is a systemic disease characterized by an extremely variable clinical presentation and long-term evolution, the development of recommendations for speeding up the diagnostic process and timely management of the condition requires a complex collaboration among several specialists. The authors consider the set of 10 tips herein presented to be an initial systematic approach for evaluating *GLA* variants in the form of an algorithm (graphical abstract), covering the most common clinical scenarios and providing better guidance for a practical diagnostic management in this setting.

## Figures and Tables

**Table 1:** Examples of *GLA* variants whose pathogenicity assessment or associated phenotype has changed over time. Variants are reported against the reference transcript NM_000169.3 (MANE Select; GRCh38). Group A lists variants for which the assessment of pathogenicity has been revised; group B lists variants that remain pathogenic and for which the associated clinical phenotype has been refined. For each variant, the reference supporting the initial assignment is reported separately from the references supporting the current interpretation, so that the two are not conflated. The revised interpretations rest on the combination of allele frequency, residual α-Gal A activity, plasma lyso-Gb3, tissue Gb3 deposition and family segregation, and not on any single criterion. Where the evidence is still discordant, the current interpretation is reported as such rather than forced into a binary pathogenic/benign assignment. α-Gal A, α-galactosidase A; FD, Fabry disease; Gb3, globotriaosylceramide; GVUS, gene variant of uncertain significance; lyso-Gb3, globotriaosylsphingosine.

*GLA* Variant	Initial Interpretation	Ref. (Initial Assignment)	Current Interpretation	Evidence Supporting the Revised Interpretation	Ref. (Revised Interpretation)
A. Variants for which the assessment of pathogenicity has been revised					
c.196G>C p.(Glu66Gln)	Pathogenic, atypical FD	N/A	Functional polymorphism; non-disease-causing	Allele frequency of 1.046% in 833 unrelated Korean individuals; residual α-Gal A activity of 19.0–30.3% in leukocytes, without the phenotype	Lee et al. [18]
c.352C>T p.(Arg118Cys)	Pathogenic, on the basis of screening studies in high-risk cohorts and of theoretical arguments on the reactivity of the cysteine residue	N/A	Likely benign; isolated conflicting reports	No Mendelian segregation with an FD phenotype in individual and family studies, high residual α-Gal A activity, and no Gb3 deposits in tissues; no association with FD features among 469,000 genotyped subjects of the UK Biobank; a single family with renal Gb3 deposits has been reported	Ferreira et al. [40]; Cerón-Rodríguez et al. [47]; Giammanco et al. [48]
c.376A>G p.(Ser126Gly)	Pathogenic/GVUS	Branton et al. [49]	Likely benign; non-disease-causing	Substantial residual α-Gal A activity and normal plasma lyso-Gb3 in carriers systematically phenotyped for FD-specific organ involvement; expert re-analysis of patients’ charts; no association with FD features in the UK Biobank cohort	Germain et al. [4]; Lau et al. [41]; Giammanco et al. [48]
c.427G>A p.(Ala143Thr)	Pathogenic, reported among the mutations identified in classically affected hemizygotes	Eng et al. [50]	Non-disease-causing or markedly reduced penetrance; conflicting interpretations in ClinVar and still open in the literature	Substantial residual activity and normal plasma lyso-Gb3 in hemizygous males; most frequent *GLA* variant in a ten-year newborn screening cohort, found in 19 of the 21 newborns carrying a benign variant, all with normal lyso-Gb3; left as GVUS by the genotype-phenotype workgroup (2-point score 27%, 13 males), with expert opinion in favor of likely benign; no association with FD features in the UK Biobank cohort	Germain et al. [4,38]; Lenders et al. [51]; Malvagia et al. [3]; Giammanco et al. [48]
c.937G>T p.(Asp313Tyr)	Pathogenic, reported among 18 new mutations in patients with the classic phenotype	Eng et al. [52]	Non-disease-causing; pseudodeficiency allele	Instability of the enzyme at the neutral pH of plasma, which lowers plasma activity while leukocyte activity remains close to normal; systematic review of the reported cases; reclassified from GVUS to benign in a diagnostic cohort of 38,242 individuals tested for FD, in which no specific clinical or biochemical FD phenotype was associated with the variant	Effraimidis et al. [17]; Boettcher et al. [53]
B. Pathogenic variants for which the associated phenotype has been refined					
c.644A>G p.(Asn215Ser)	Pathogenic, cardiac involvement described in single asymptomatic or mildly affected hemizygotes with residual activity	Eng et al. [52]	Late-onset cardiac phenotype, systematically characterized (pathogenicity unchanged)	Phenotypic characterization in hemizygous males and heterozygous females in registry and cohort studies; in the UK Biobank cohort, p.(Asn215Ser) was the only non-classic variant associated with the FD phenotype score	Germain et al. [43]; Giammanco et al. [48]
c.640-801G>A (legacy: IVS4+919G>A; c.639+919G>A; c.936+919G>A)	Pathogenic, rare cardiac variant identified in a single hemizygote with 9.1% residual activity and absent in 100 unaffected Japanese males	Ishii et al. [46]	Pathogenic, later-onset cardiac phenotype with incomplete penetrance; clinical relevance debated	Newborn screening in Taiwan showed an incidence of about 1 in 1250 to 1 in 1600 males, far above the expected prevalence of FD; a subsequent genetic epidemiological study did not support the variant as a frequent cause of overt heart disease, and a multifactorial rather than monogenic model was proposed	Hwu et al. [54]; Lin et al. [55]; Chiang et al. [56]

**Table 2:** Complete list of experts’ tips for assessment of *GLA* variants in Fabry disease. All tips are the expert opinion of the panel, informed by the cited literature; they do not derive from a formal grading of the evidence.

Items	Experts’ Tips	
Diagnostic Setting	1	Verify whether the diagnostic procedure is started following a clinical sign/symptom suggestive of FD, if it is part of an extended or selective screening (i.e., prenatal, neonatal, familial), or if it is an incidental finding during genetic assessment for another Mendelian condition.
Family History	2	Extend testing to relatives as directed by the pedigree and by the confirmed familial variant, rather than by a fixed degree of relatedness. Family members who test positive should follow the diagnostic procedure of the proband.
Metabolic Profile	3	Carry out a α-Gal A assay on DBS for screening and on cells (i.e., leukocytes, fibroblasts) for confirmation and conduct lyso-Gb3 measurement in plasma.
Renal Profile	4	Perform kidney biopsy and histological examination in subjects with progressive renal disease of unknown origin along with immunohistochemical investigation for Gb3 deposits and electron microscopy; lamellar inclusions (zebra bodies) are characteristic but not specific, being also seen in drug-induced phospholipidosis. In asymptomatic subjects, evaluate whether to perform a kidney biopsy only in specific cases and based on available evidence of other FD-related organ involvement.
	5	Perform repeated measurements of (a) eGFR with creatinine and cystatin C, checking for hyperfiltration, (b) spot ACR/PCR, (c) urinary sediment, (d) renal ultrasound to check for the presence of parapelvic cysts.
Cardiological Profile	6	Consider endomyocardial biopsy, in specialized centers, in patients with cardiac signs or symptoms suggestive of FD when the heart appears to be the only organ involved, non-invasive evaluation including CMR is inconclusive, and the result is expected to change management.
	7	Perform measurement of serological biomarkers and evaluation of heart function by ECG, Holter, echocardiogram, and CMR to check for imaging signs of FD.
Neurological Profile	8	Carry out a thorough case history to assess the presence of other systemic signs and symptoms of FD and complete neurological examination to select subjects to be referred for further instrumental investigations.
Systemic Signs and Symptoms	9	Assess the presence of early-onset systemic symptoms (e.g., GI alterations, acroparesthesias), and carry out differential diagnosis.
	10	Assess the presence of early-onset pathognomonic or highly suggestive signs of FD, i.e., cornea verticillata and angiokeratomas, and of less specific signs that support multiorgan involvement, i.e., hypoacusis and hypohidrosis/anhidrosis.

## Data Availability

No new data were created or analyzed in this study. Data sharing is not applicable to this article.

## Abbreviations

The following abbreviations are used in this manuscript:

FD	Fabry disease
LSD	lysosomal storage disorder
α-Gal A	α-galactosidase A
Gb3	globotriaosylceramide
lyso-Gb3	globotriaosylsphingosine
ERT	enzyme replacement therapy
VUS	variant of uncertain significance
ACMG	American College of Medical Genetics and Genomics
AMP	Association for Molecular Pathology
P	pathogenic
LP	likely pathogenic
LB	likely benign
B	benign
NGS	next-generation sequencing
AF	allele frequency
DBS	dried blood spot
GFR	glomerular filtration rate
CKD	chronic kidney disease
ESRD	end-stage renal disease
eGFR	estimated glomerular filtration rate
β2M	beta 2-microglobulin
NGAL	neutrophil gelatinase-associated lipocalin
PC	parapelvic cysts
ISGFN	International Study Group of Fabry Nephropathy
ACR/PCR	albuminuria/proteinuria
QoL	quality of life
LV	left ventricular
LVH	left ventricular hypertrophy
NT-proBNP	brain natriuretic propeptide
S1P	sphingosine-1-phosphate
hs-cTnI and hs-cTnT	high-sensitivity cardiac troponin
ECG	electrocardiogram
AV	atrioventricular
AFib	atrial fibrillation
RBBB	right bundle branch block
LBBB	left bundle branch block
CMR	cardiac magnetic resonance
FOS	Fabry Outcome Survey
LVOTO	LV outflow tract obstruction
RV	right ventricle
RVH	RV hypertrophy
LGE	late gadolinium enhancement
PET	positron emission tomography
EMB	endomyocardial biopsy
PNS	peripheral nervous system
CNS	central nervous system
SFN	small fiber neuropathy
SVD	small vessel disease
WMHs	white matter hyperintensities
SBI	small vessel infarcts
EPVs	enlarged perivascular spaces
QSART	Quantitative Sudomotor Axon Reflex Tests
GI	gastrointestinal
IBS	irritable bowel syndrome
